# Realizing the Wishes of Terminal Patients: Caregiving Transport Efforts for End of Life in the Kuji Area of Japan

**DOI:** 10.1089/pmr.2020.0113

**Published:** 2021-02-26

**Authors:** Mizunori Yaegashi, Koki Otsuka, Kasumi Nitta, Chihiro Tono, Yukihiro Minagawa, Toru Yoshida, Hidenobu Kawamura

**Affiliations:** ^1^ Department of Surgery, School of Medicine, Iwate Medical University, Shiwa, Iwate, Japan.; ^2^ Department of Surgery, Iwate Prefectural Kuji Hospital, Kuji, Iwate, Japan.; ^3^ Department of Nursing, Iwate Prefectural Kuji Hospital, Kuji, Iwate, Japan.; ^4^ Department of Surgery, Iwate Prefectural Miyako Hospital, Miyako, Iwate, Japan.

**Keywords:** cardiopulmonary resuscitation, do-not-attempt-resuscitation, do not resuscitate orders, instruction of physician, terminal patients

## Abstract

***Background:*** There are some restrictions in Japan regarding end-of-life care. For example, only physicians can legally issue death certificates. By law, ambulance staff members perform cardiopulmonary resuscitation (CPR) for all patients with cardiopulmonary arrest (CPA). Therefore, it is difficult to transport patients to hospitals without CPR, even in cases of terminal patients with do-not-attempt-resuscitation (DNAR) order. Furthermore, there is no 24-hour home care nursing system in our area. Therefore, terminal patients are unable to spend their last moments at their home in the Kuji area.

***Objective:*** To design a system in which terminal patients who wish to spend their final moments at their home can be transported to the hospital without CPR after at-home CPA and a system to avoid confusion between ambulance staff and family members using instructions provided by the physician.

***Setting/Subjects:*** The subjects were terminal patients with DNAR order who wanted to stay at home. The instruction to not perform CPR after CPA was created as a document by physicians. Patient information was shared with the fire department; patients were transported to our hospital without CPR after at-home CPA.

***Results:*** In total, 26 patients died during the study period; eight received emergency transport to the hospital without CPR after CPA. CPR was not performed for any patient.

***Conclusion:*** A system transporting terminal patients without CPR after CPA was necessary in our area. This instruction allows terminal patients to spend their last moments where they wish and avoids unwanted CPR and troubles after CPA.

## Introduction

With the aging population and a growing number of cancer patients, cancer has the highest number of deaths in Japan.^1^ Therefore, end-of-life care that respects the desired lifestyle of terminal patients with diseases such as cancer is extremely important.^2,3^ Medical and health care in Japan cannot meet these needs, and places for end-of-life care cannot be secured. As efforts based on advance care planning^4^ defined in Europe and North America are increasing, “The Practice Guidelines for Process of Decision-Making Regarding Treatment in the End-of-Life Care” was prepared in Japan in 2018.^5^ The final stage of life according to this guideline is defined as the end of life during the terminal phase of a disease. To provide medical care to patients so that they can stay where they choose, efforts should involve medical staff, caregivers, communities, and the federal government.

According to a 2019 report by the ministry of health, labor, and welfare, Kuji Hospital ranked low in terms of physician distribution for medical care for general practice in Japan and has a shortage of physicians.^6^ There are some restrictions in Japan regarding the end-of-life care as follows: (1) death certification is legally performed only by physicians; (2) there is no general practitioner and nurse practitioner system in Japan because of which some patients do not have access to the practitioner they visited every day in the night and weekends (i.e., physician who is easily accessible is from an emergency hospital in our area); (3) emergency ambulance transportation to hospital is completely free regardless of nationality, and this cost is covered by the national tax system; and (4) there is no legal validity for an advance directive that makes a doctor's indication essential at the time. In addition, there is no 24-hour home care nursing system in the Kuji area, and it is difficult to provide home visits for end of life. Therefore, regardless of patient's wish, all terminal patients spend their last moments at the hospital. When terminal patients have a do not attempt resuscitation (DNAR) order go into cardiopulmonary arrest (CPA) at home or a facility of their choice, cardiopulmonary resuscitation (CPR) is performed by paramedics, and the patients are transported to our hospital because we have no physicians who can provide home visits. Ambulance staff members have to perform CPR for all patients with CPA by law, even in cases of terminal patients with DNAR. Consequently, DNAR causes confusion between ambulance staff members and family members after CPA at their home, and instructions from physicians are necessary during such emergencies in Japan. A system of transporting terminal patients to our hospital without CPR after CPA from their home could be a valuable option in our area.

The purpose of this study was to design and establish a system wherein terminal patients who wished to spend their final moments at their home are transported to the hospital without CPR after at-home CPA and a system to avoid confusion between ambulance staff members and family members using instructions provided by the physician, which included working with our hospital, the fire department, and the city and their respective staff members. We report this policy in our community.

## Methods

### Design, setting, and population

The subjects were terminal patients who visited Iwate Prefectural Kuji Hospital between November 2018 and January 2020. All patients included in this study were inpatients or outpatients of our hospital. Terminal patients were defined as follows: (1) patients at the terminal stage of malignant disease wherein the prognosis is several days to two to three months, (2) patients regardless of the existence of chronic disease (i.e., cirrhosis, cardiac insufficiency, chronic obstructive pulmonary disease, and sequelae of cerebrovascular disease) who have gradually debilitated and will die, or (3) patients whose prognosis is expected to be less than one year by the attending physician. When a patient with predictable prognosis in the final stage does not wish for CPR at CPA and the patient or a family member/guardian who can decide on behalf of the patient requests to stay at home or a facility other than the hospital, the following protocol was applied. The study protocol was approved by the ethical committee of Iwate Prefectural Kuji Hospital (There is no approval number; approval date: September 19, 2019). Verbal and written informed consent was taken from the participants. This study was conducted in accordance with the Declaration of Helsinki as revised in 2013. We orally asked family members and relatives who visited our hospital after the death of patients about their impressions of this policy.

### Protocol

**FIG. 1.**
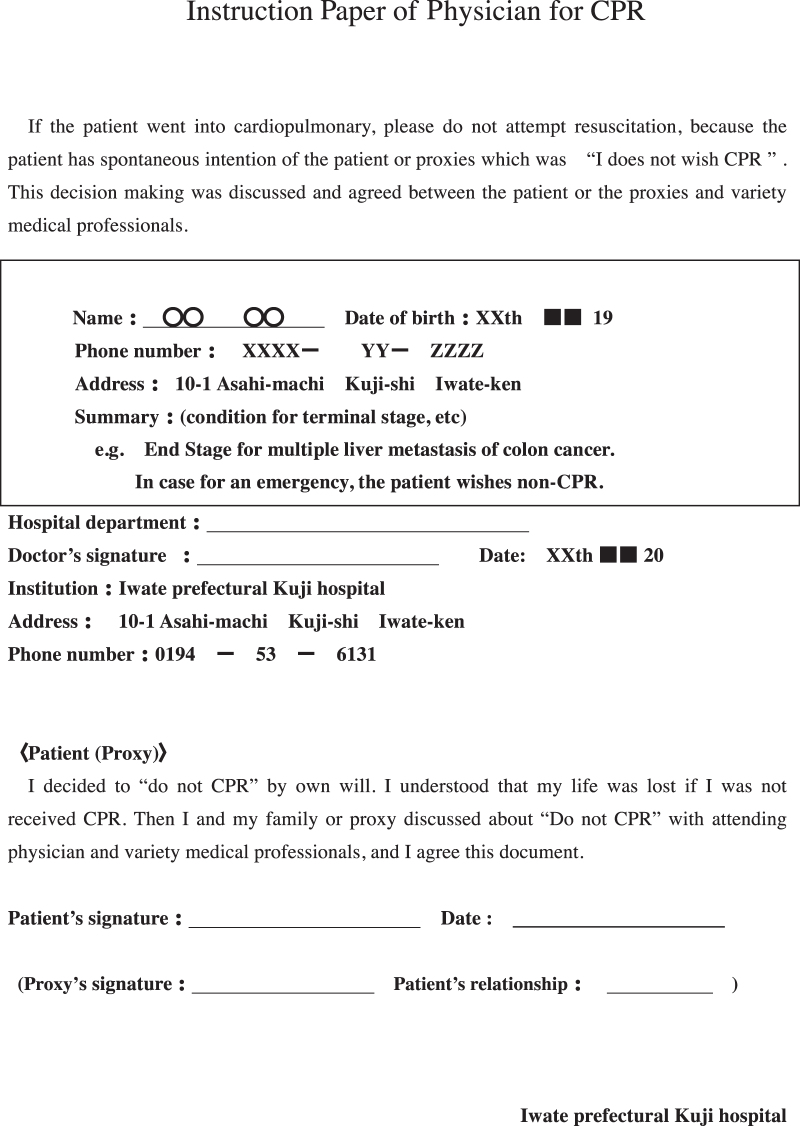
Physician's instructions for CPR. CPR, cardiopulmonary resuscitation.

**FIG. 2.**
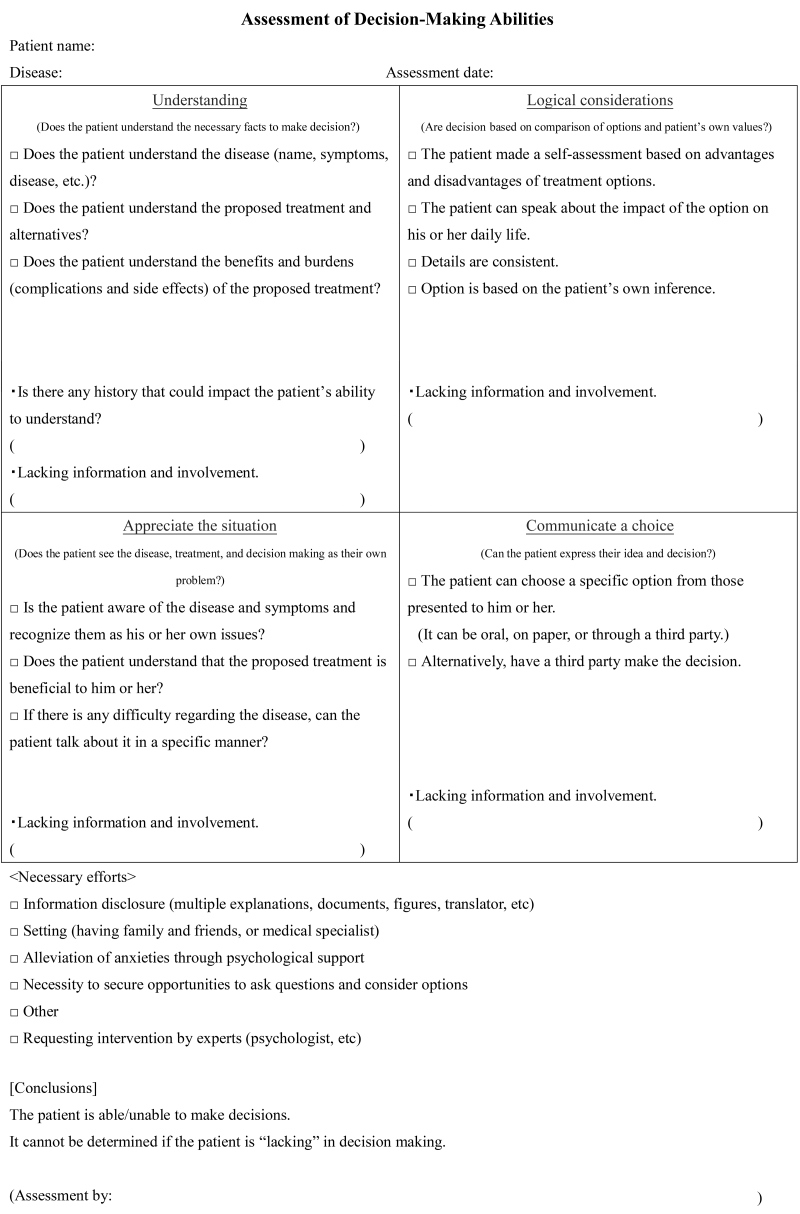
Assessment of decision-making abilities. As shown in the upper part of the image, understanding, awareness, ethical considerations, and statement are comprehensively used for assessment. In the lower part, potentials to improve abilities are listed for use in cases wherein the patient lacks decision-making skills.

**FIG. 3.**
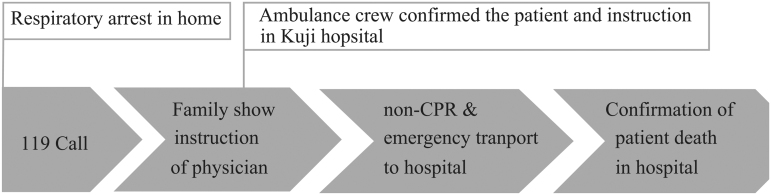
Flow from cardiac arrest to confirmation of death. If a patient goes into cardiac arrest at home or a facility, the family or other party called 119. They presented the physician's instructions for cardiopulmonary resuscitation to the arriving paramedics. Paramedics confirmed the physician's instruction and patient information with the emergency department of Kuji Hospital and transported the patient to the hospital in an ambulance. The patient's death was confirmed at the hospital.

The palliative care team and the attending physician provided a written detailed explanation of the patient's medical conditions to the patient who then confirmed the patient's wish for DNAR in writing. If patients did not have the capacity to make decisions, a family member or guardian was given an explanation of the patient's medical conditions. According to the suggestion for CPR as an emergency activity as per patients' will,^7^ attending physicians prepared a document outlining the physician's instructions for CPR (Fig. 1). The palliative care team and ethical committee examined the validity of this instruction. Whether patients were able to make their own decision was assessed based on the evaluation points by Grisso and Appelbaum^8^ (Fig. 2). After preparing the instruction, the hospital shared the patient information with the medical control room of the Kuji fire department. When patients went into CPA at home or a facility, the family or medical care provider (i.e., visiting nurses and care managers) called 119 (Japan's direct dial emergency number) to send paramedics to the home or the facility. The family or the paramedic presented the physician's CPR instructions to the paramedics, who then contacted Kuji Hospital to confirm the instruction with the physician. After confirmation, patients were transported in an ambulance without CPR to Kuji Hospital, and the physician confirmed the death in the emergency department (Fig. 3). The patient's intention for DNAR was confirmed repeatedly during hospital visits, admission, and discharge. If one year passed since the physician's directive was issued, the intention of DNAR was confirmed again, and the document was updated. Then, we requested home care nursing support from medical areas in neighboring cities or prefectures because there is no 24-hour home care nursing system in Kuji city.

For the mentioned protocol, a medical control meeting was held between Kuji Hospital, Kuji City Hall, and the fire department at our medical zone. At this meeting, an emergency protocol was approved for patients who expressed DNARs.

## Results

Thirty-seven terminal patients who wished to spend their last moments at home were screened by their physicians. Among 37 patients approached in this study, all patients themselves or their families or agents expressed their intention to participate. Then, all patients were provided with the documents of physician's non-CPR instruction. Among 37 patients, 19 patients were given an explanation of DNAR and expressed their intention to sign DNAR. The remaining 18 patients had no decisional capacity. Hence, a family member or guardian was given an explanation of DNAR and decided to sign DNAR assuming their intention whether they were fine. The involvement of palliative care team intervention was required in all cases who participated in this study to work with the fire department and the city and their respective staff members, and the ethical committee assessed the validity of the instruction. The mean age of patients was 82.9 (range, 57–101) years, and the disease was malignant in 25 (67.6%), chronic in 7 (18.9%), and senile decay in 5 (13.5%) patients. Twenty-six patients (70.3%) were at home and 11 (29.7%) remained at facilities. Among the 37 patients, 26 died during the study period (Table 1). Among these, 21 (80.8%) and 5 (19.2%) patients had malignant and benign diseases, respectively.

**Table 1.** Cases in Which Patients Died after the Do Not Attempt Resuscitation Instruction Was Provided

Patient no.	Age, years	Disease	Medical care zone of Kuji	Period until death (days)	Institution^a^	Result^b^
1	82	Renal pelvic cancer	Yes	10	Home	Home
2	76	Pancreatic cancer	No	42	Home	Hospital
3	57	Gastric cancer	No	224	Home	Hospital
4	91	Gastric cancer	Yes	196	Home	Home
5	89	Mesothelial sarcoma	No	26	Home	Home^c^
6	68	Gastric cancer	Yes	7	Home	Hospital
7	68	Lung cancer	No	1	Home	Home
8	84	Bile duct carcinoma	Yes	104	Home	Home
9	95	Colon cancer	Yes	12	Facility	Facility
10	66	Lung cancer	Yes	7	Home	Home
11	96	MDS	Yes	22	Home	Hospital
12	86	Bile duct carcinoma	Yes	101	Facility	Hospital
13	94	COPD	Yes	20	Home	Hospital
14	79	Pancreatic NEC	Yes	11	Home	Hospital
15	86	Lung cancer	Yes	13	Home	Hospital
16	79	Hepatic cancer	Yes	18	Home	Hospital
17	86	Pancreatic cancer	Yes	16	Home	Hospital
18	88	Senile decay	Yes	7	Facility	Hospital
19	69	COPD	Yes	17	Home	Home
20	69	Hepatic cancer	Yes	6	Home	Hospital
21	81	Hemangiosarcoma of the liver	Yes	29	Home	Hospital
22	71	Pulmonary fibrosis	Yes	35	Facility	Facility
23	82	Pancreatic cancer	Yes	23	Home	Hospital
24	87	Pancreatic cancer	Yes	25	Home	Hospital
25	79	Colon cancer	Yes	22	Home	Home
26	76	Lung cancer	Yes	10	Home	Hospital

^a^ Location where patients wished to spend their last moments.

^b^ Location where the patient suffered a cardiac arrest.

^c^ Autopsy.

COPD, chronic obstructive pulmonary disease; MDS, myelodysplastic syndrome; NEC, neuroendocrine carcinoma.

Eight (30.8%) patients went into CPA at home or a facility and received emergency transport to our hospital without CPR. Their deaths were confirmed at the hospital. There was no trouble in cooperation between family, facility staff, paramedics, and hospital staff (i.e., physicians or nurses) from the moment of CPA emergency notification to transportation to our hospital. DNAR notification in all patients' electronic file was smoothly confirmed whether the patient had signed a DNAR, when ambulance staff was connected with our hospital from the scene. Then, physician of the hospital indicated the ambulance staff not to perform CPR. Sixteen (61.5%) patients were rehospitalized and died during hospitalization. One patient's death (3.8%) was confirmed by a part-time physician at a facility. Another patient (3.8%) was not transported and was autopsied because of rigor mortis. The median time from the instruction to the confirmation of death for the 26 deceased patients was 17 (range, 1–224) days. The deaths of all 17 patients who died at the hospital after being readmitted and at the facility were confirmed without CPR.

Family members of six of eight (75.0%) patients who received emergency transport with non-CPR were asked about their impressions of this policy after the death of patients. We found that all family members appreciated the policy because they were able to spend their last moments where they wanted, even if it was for a short period.

## Discussion

For end-of-life patients, it is important to respect the dignity of patient's life that health care providers comprising interprofessional teams intervene during the early stages and support their desired lifestyle and medical service.^9,10^ Among the Japanese, the idea of a good death often shares the same traits: alleviation of physical and mental pain, not putting a burden on one's family or others, and to die where they choose.^11^ Current Japanese law stipulates the standard of emergency care provided by paramedics^12^ as per this law, it is difficult to transport patients with CPA to hospitals without CPR, even in cases of terminal patients with DNARs. Consequently, DNAR causes confusion between paramedics and family members, and instructions from physicians are necessary during emergency in Japan.^13^ Nakagawa et al.^14^ reported that most hospitals have experienced patients with DNARs, yet not many hospitals have a guideline for DNARs. When patients with DNARs go into CPA at their home, it is unclear whether CPR should be performed. Hence, understanding DNARs and the formulation of guidelines by each municipality in Japan is necessary.^1^

We created a unique policy to provide an option for terminal patients to spend their last moments where they wished. Our policy is a system that uses the physician's instructions for CPR, and the patient's intention of DNAR is shared between our hospital, the fire department, the city, and their respective staff members. Moreover, it is designed to avoid troubles after at-home CPA and transportation to our hospital without CPR. To the best of our knowledge, this policy is the first to be reported in Japan. We first assumed our subjects would be terminal cancer patients. However, we discovered similar needs for noncancer patients and those at other facilities. Furthermore, 60% patients could not manage their deteriorating health and sudden change in symptoms. These patients were repeatedly hospitalized to manage their symptoms and ultimately died during hospitalization. This clarified the lack of home visiting physicians. However, 30% patients died at the locations they chose and avoided unwanted invasive resuscitation attempts. There was no trouble in cooperation between family, facility staff, paramedics, and hospital staff (i.e., physicians or nurses) from the moment of CPA emergency notification to transportation to our hospital, indicating the success of this community effort. DNAR notification in the patient's electronic file was specified to allow fast confirmation whether the patient has a DNAR or not. Because we provided patient information to the fire department, home care nurses, and care support experts, cooperation among them was smooth.

There are several limitations of this study. The study period was short, and the number of patients was small. In this study, all patients or their family member or guardian indicated their intention to participate, but it is possible that patients may not agree with this policy in the future. Thus, problems associated with this policy may not be identified. For instance, emergency patient and terminal patient may overlap in an area with only one ambulance available. We also need to assess the emotional experience of physicians, paramedics, associated staff, and families who experienced this policy. In addition, we need to strengthen cooperation and renew information of this policy among our community and local residents.

## Conclusion

There are no physicians for home visit as well as a 24-hour home care nursing system in our area. For terminal patients to spend their last moments in a familiar place, we developed a system wherein patients are transported to the hospital without CPR upon their death using the physician's CPR instructions. The need for this policy in our community is widespread, and such requests are expected to increase in future. Thus, we must establish end-of-life measures for each community by responding to these challenges.

## Abbreviations Used

COPD: chronic obstructive pulmonary disease
CPA: cardiopulmonary arrest
CPR: cardiopulmonary resuscitation
DNAR: do not attempt resuscitation
MDS: myelodysplastic syndrome
NEC: neuroendocrine carcinoma
